# The impact of nonsyndromic cleft lip with or without cleft palate on oral health-related quality of life

**DOI:** 10.1590/1678-7757-2017-0145

**Published:** 2018-03-26

**Authors:** Maria Augusta Ramires da Silva, Isis de Fátima Balderrama, Ana Paula Wobeto, Renata Iani Werneck, Luciana Reis Azevedo-Alanis

**Affiliations:** 1 Pontifícia Universidade Católica do Paraná, Escola de Ciências da Vida, Curitiba, PR, Brasil; 2 Universidade de São Paulo, Faculdade de Odontologia de Bauru, Bauru, SP, Brasil

**Keywords:** Quality of life, Cleft lip, Dentistry, Oral health

## Abstract

Nonsyndromic cleft lip with or without cleft palate (NSCL±P) compromises oral health, leading to missing or malformed teeth, and hampering oral hygiene. Apart from anatomic damages, NSCL±P also culminates in an impact on the routine quality of life with social privation and psychological embarrassment.

**Objective:**

To evaluate the impact of NSCL±P on oral health-related quality of life.

**Material and Methods:**

The study was classified as exploratory and descriptive, with quantitative approach. Patients with NSCL±P treated between August 2013 and September 2014 at the Cleft Lip and Palate Integral Care Center (CAIF), Curitiba, Brazil, were invited to participate. Age and sex-related data were collected, as well as level of education, financial income, type of orofacial cleft, use of orthodontic and prosthetic appliances, and number of previous orofacial surgeries. Selected patients were asked to answer the Oral Impact on Daily Performance (OIDP) questionnaire designed to measure the impact of oral health on daily performances.

**Results:**

The sample was composed of 103 (44.59%) women and 128 (55.41%) men, with mean age of 19.74 ± 10.20 (7–65) years. The OIDP values ranged from 1 to 175 in 114 (49.35%) patients (mean: 22.38), whereas 117 patients (50.65%) presented total OIDP value equal to zero. High negative impact of NSCL±P on daily performances was detected when associated with the female sex (p=0.037). Daily performances related to phonetics (OIDP2; 2.63) and aesthetics (OIDP5; 2.48) presented the highest average values when compared to other daily performances, except OIDP6. The main symptoms and reported oral problems comprised the aesthetic dissatisfaction.

**Conclusions:**

Almost half of the patients evaluated in this study showed negative impact of NSCL±P in the performance of daily activities.

## Introduction

Cleft lip with or without cleft palate is a common congenital disorder^18^ resulting from the lack of merging between the embryonary structures that precede the formation of lip and palate^6^. About 70% of oral clefts occur as a nonsyndromic form, and the remaining 30% are associated with Mendelian disorders or chromosomal, teratogenic and sporadic conditions^12^. The prevalence of nonsyndromic cleft lip with or without cleft palate (NSCL±P) varies according to ethnicity and geographic position; in Brazil, it is estimated at 0.99 (95% confidence interval, 9.6–10.2) *per* 1,000 live births^13^ ^,^ ^17^. NSCL±P etiology is related to a complex interplay between environmental exposures, genetic, and epigenetic factors^9^ ^,^ ^14^.

Clinically, NSCL±P compromises oral health, leading to missing or malformed teeth, and hampering oral hygiene. Moreover, the relation between dental loss and oral cleft is still being discussed, indicating that critical factors in the pathogenesis of the cleft lip are also critical for the odontogenesis, thus showing possible different subphenotypes^3^. Apart from anatomic damages, NSCL±P also culminates in an impact on the routine quality of life with social privation and psychological embarrassment^22^.

Oral health clinical conditions may have an important impact on oral health-related quality of life (OHQoL). According to the World Health Organization (WHO), quality of life consists in the perception of the human being in relation to cultural and social values of the environment, as well as objectives, expectations, and concerns for life^23^. Social- and health-based tools were developed to measure the quality of life by physical, psychological and social parameters. Some of these tools were adapted for dental investigations, allowing to detect dentomaxillofacial alterations that possibly interfere within the daily routine of patients^2^ ^,^ ^8^ ^,^ ^15^ ^,^ ^16^. In this context, the Index of Oral Impacts on Daily Performances (OIDP), based on the international classification of the WHO for disabilities and deficiencies^23^, was developed by Adulyanon and Sheiham^2^ (1997), and validated for application in Portuguese language by Abegg, et al.^1^ (2015). Specifically, the OIDP aims to evaluate the impact of oral health during daily performances that may potentially affect the quality of life. Thus, the application of this tool adds valuable information to clinical surveys, enhancing related researches and further treatment planning^4^.

Because NSCL±P patients may present social, phonetic and aesthetic disorders, as well as hampered oral hygiene, our study aimed to evaluate the impact of nonsyndromic cleft lip with or without cleft palate on oral health-related quality of life.

## Material and methods

This study was approved by the Institutional Review Board under the protocol number 3538/09.

The current study design is classified as exploratory and descriptive, with quantitative approach.

The sample consisted of patients with NSCL±P treated between August 2013 and September 2014 at the Cleft Lip and Palate Integral Care Center (CAIF), Curitiba, Southern Brazil. The sample size was calculated with 95% confidence interval and 6% maximum error limit, assuming 50% (*p*) of potential impact of NSCL±P on OHQoL. Thus, the sample size was estimated in 231 patients. Syndromic patients were not included in the study. Patients and relatives that did not wish to participate in the research were not included in our study, as well as patients that were unable to answer the questionnaire. The remaining patients were informed about the research aims and design, and signed an informed consent form. If the patient was below 18, the respective relatives signed the form.

After the selection criteria, a sample of 231 patients with NSCL±P was screened. Personal data related to sex, age, level of education, financial income, NSCL±P (extension and location), previous orofacial surgeries, and the use of orthodontic or prosthetic appliances were retrieved from each patient.

The patients were asked to answer the OIDP questionnaire. It was applied and answered in a separate room at CAIF, which assured comfort and privacy for the patients. Two trained examiners clearly explained and read each of the provided questions to each patient. All the answers comprehended the patient's personal data related to the previous six months.

Eight different performances were addressed by the questionnaire: Physical performances: OIDP1 – Routine eating; OIDP2 – Clear speaking; OIDP3 – Oral Hygiene; Psychological performances: OIDP4 – Sleeping and relaxing; OIDP5 – Smiling and showing teeth without becoming embarrassed; OIDP6 – Maintaining stable emotional status; Social performances: OIDP7 – Working; OIDP8 – Contacting people.

The questionnaire comprehended four questions related to the eight performances. The first question aimed to detect the frequency of limitations on daily performances due to the NSCL±P. In case of a positive answer, the patient was requested to indicate the level of frequency using a score from 1 to 5. In case of a negative answer, the patient was guided to the next performance. The second question aimed to measure the severity of the limitation, according to the patient's view. At this stage, scores from 0 to 5 were applied. The third question approached the main symptoms reported by NSCL±P patients, such as pain, discomfort, and functional and aesthetic limitations. The fourth question aimed to assess the relation between oral and dental complications from NSCL±P with limitations on daily performances.

The values of each OIDP were calculated from the first and second questions, according to the literature^10^ ^,^ ^15^. Thus, the value of each OIDP varied from 0 to 25. Additionally, the total OIDP value for the eight performances was calculated from each patient, ranging from 0 to 200. The final OIDP value was dichotomized based on the mean value of the sample (22.38): high negative impact of NSCL±P on daily performances ≥22.38; low negative impact <22.38.

We performed the statistical analyses using SPSS 22.0 (IBM; New York, NY, USA) software. The correlation between the total OIDP value and age was performed through Pearson's correlation coefficient, and Z test (difference between two proportions). Chi-square test was used to associate the total OIDP value with sex, financial income, NSCL±P (extent and site), number of previous orofacial surgeries, and use of orthodontic/prosthetic appliances. We performed the comparison between OIDP values using Student's t test for paired samples.

For all tests, a significance level of 5% (p<0.05) with 95% confidence intervals was assumed.

## Results

The age range of 231 patients with NSCL±P varied between 7 and 65 years (mean: 19.74±10.20). Women comprehended 44.59% (n = 103) of the sample, while 55.41% (n = 128) were men. Regarding the type of oral cleft, 22 (21.36%) women showed NSCL, 12 (11.65%) showed NSCP, and 66 (64.07%) showed NSCLP. Table 1 shows the sample distribution according to the studied variables.

**Table 1:** Sample distribution according to the studied variables

Variables		n (%)
Sex	Female	103 (44.59)
	Male	128 (55.41)
	Incomplete formal education	119 (51.51)
Level of education	Complete formal education (high-school or undergraduation)	112 (48.49)
	<2 minimum wages	167 (72.29)
Financial income	From 2 to 20 minimum wages	60 (25.97)
	Not reported	4 (1.74)
	Anterior to the foramen	70 (30.30)
Extent of NSCL ± P	Overlapping the foramen	138 (59.74)
	Posterior to the foramen	17 (7.35)
	Rare cleft lesions	6 (2.61)
	Cleft lip only	50 (21.64)
Site of NSCL ± P	Cleft palate only	20 (8.66)
	Cleft lip and palate	154 (66.67)
	Not reported	7 (3.03)
	≤5	153 (66.23)
Number of previous orofacial surgeries	≥6	74 (32.03)
	Not reported	4 (1.74)
	Removable appliance	13 (5.63)
Orthodontic appliances	Fixed appliance	119 (51.51)
	None	92 (39.83)
	Not reported	7 (3.03)
	Present	37 (16.02)
Prosthetic appliances	Absent	187 (80.95)
	Not reported	7 (3.03)

A total of 114 patients (49.35%) presented total OIDP value ranging between 1 and 175 (mean value: 22.38±27.66). Thirty-one patients (27.19%) showed high negative impact of NSCL±P on daily performances (OIDP≥22.38), while eighty-three patients (72.81%) presented low negative impact of NSCL±P on daily performances (OIDP<22.38). A total of 117 patients (50.65%) presented total OIDP value equal to zero.

The high negative impact of NSCL±P on daily activities (OIDP≥22.38) was significantly associated with the female sex (p=0.037).

No significant association was detected between the total OIDP value and the age, level of education, financial income, NSCL±P (extent and site), use of orthodontic and prosthetic appliances, and number of previous orofacial surgeries (p>0.05).

The mean values for the eight performance scores, and the main oral and dental complications from NSCL±P are expressed in Table 2.

**Table 2:** Mean values and standard deviation (SD) of the Oral Impact on Daily Performance (OIDP), and the oral/dental symptoms and complications from NSCL±P on daily performances

Daily performances	Mean OIDP ± SD	N (%)	Oral/dental symptoms	N (%)	Oral/dental complications	N (%)
**Physical Performances**						
OIDP1. Routine eating	1.04 ± 3.90	20 (8.65)	Function limitation	6 (30.00)	Hampered breathing	4 (22.05)
			Discomfort	5 (25.00)	Missing teeth	3(15.79)
OIDP2. Clear speaking	2.63 ± 5.40	58 (25.10)	Function limitation	34 (59.65)	Orofacial malformation	7(17.50)
			Not identifiable	12 (21.05)	Hampered breathing	3 (7.50)
OIDP3. Oral hygiene	0.99 ± 4.17	15 (6.49)	Pain	7 (50.00)	Tooth pain	3 (23.08)
			Function limitation	3 (21.43)	Orofacial malformation	2 (15.38)
**Psychological Performances**						
OIDP4. Sleeping and relaxing	0.58 ± 2.87	13 (5.62)	Discomfort	6 (46.15)	Hampered breathing	3 (27.28)
			Aesthetic dissatisfaction	4 (30.77)	Orofacial malformation	1 (9.09)
OIDP5. Smiling and showing teeth without becoming embarrassed	2.48 ± 6.08	44 (19.04)	Aesthetic dissatisfaction	34(79.07)	Missing teeth	10 (27.78)
			Not identifiable	6 (13.95)	Orofacial malformation	9 (25.00)
OIDP6. Maintaining stable emotional status	1.83 ± 5.28	40 (17.31)	Not identifiable	18 (50.00)	Orofacial malformation	4 (14.81)
			Aesthetic dissatisfaction	12 (33.33)	Hampered breathing	3 (11.11)
**Social Performances**						
OIDP7. Working	0.75 ± 3.85	13 (5.62)	Aesthetic dissatisfaction	8 (61.54)	Orofacial malformation	5 (38.46)
			Function limitation	2 (15.38)	Displaced teeth	2 (15.38)
OIDP8. Contacting people	0.74 ± 3.74	14 (6.06)	Aesthetic dissatisfaction	8 (57.14)	Orofacial malformation	5 (35.71)
			Not identifiable	3 (21.43)	Displaced teeth	4 (28.57)

Mean OIDP2 (2.63) and OIDP5 (2.48) values were significantly higher than the other OIDP values (p<0.05), except for OIDP6 (1.83). A positive, however discrete, significant correlation was found between OIDP2 and OIDP5 (r=0.172; p = 0.009) (Table 2).

A total of 58 (25.10%) and 44 (19.04%) patients revealed speaking (OIDP2) and smiling (OIDP5) limitations, respectively. Considering OIDP2, 34 patients (59.65%) indicated function limitation as the main symptom, while seven (17.50%) indicated orofacial malformation (Table 2).

Mean OIDP5 (p = 0.023) and OIDP6 (p = 0.035) values for patients who use orthodontic and prosthetic appliances were 1.081 and 0.729 compared to 2.742 and 2.036 for patients who do not use appliances, respectively.

## Discussion

The impact of OHQoL has been extensively explored in the medical literature^2^ ^,^ ^4^ ^,^ ^5^ ^,^ ^7^ ^,^ ^8^ ^,^ ^15^ ^,^ ^16^ ^,^ ^19^. NSCL±P patients often present phonetic, aesthetic and masticatory limitations, as well as difficulties for oral hygiene and social activities^18^ ^,^ ^22^, compromising the quality of life. Based on that, our study aimed to investigate the impact of NSCL±P on OHQoL. NSCL±P showed negative impact on the performance of daily activities of approximately half of the evaluated sample, and of these, 27.19% showed high negative impact. The high negative impact of NSCL±P on daily activities was associated with the female sex. The most affected performances were OIDP2 – clear speaking, and OIDP5 – smiling and showing teeth without becoming embarrassed.

The high frequency of patients experiencing the negative impact of NSCL±P on the performance of daily activities in this study is in accordance with a previous investigation^22^. The physical changes caused by NSCL±P, such as dental loss and deformity of the face, as well as difficulties in speech, chewing and swallowing, can change the socio-affective relations of patients, bringing many social challenges to these patients and reflecting in their quality of life.

In this study, the female sex was associated to the high negative impact of NSCL±P on daily activities. The fact that women are more concerned with oral health than men may explain this result because they have a different perception of oral health compared to men^5^.

In this study, the OIDPs 2 and 5 showed the highest mean values when compared to other daily performances. Moreover, a larger number of patients showed negative impact on OIDPs 2 and 5 compared to the other OIDPs. These data confirm the impressions during data collections: the patients showed a direct relationship between these two performances, reporting that they did not smile and did not speak because of dissatisfaction with appearance or deformity of his/her mouth or face. The deformity of the mouth or face was a major dental problem mentioned by patients in both performances. In addition, out of 44 patients with negative impact on OIDP5, 10 (27.78%) indicated that the main reason for this difficulty was missing tooth. Mendonça, et al.^11^ (2010) reported that 41.3% of patients with missing teeth had difficulty in smiling.

In this study, although only 15 (6.49%) patients reported problems to hygienize the teeth (OIDP3), 50% of them reported feeling pain when performing this function. This result agrees with Gomes and Abbeg^5^ (2007), who reported that the pain was significant and impacted the quality of life of patients, although they did not evaluate patients with NSCL±P. According to Suliman, et al.^19^ (2012), the evaluation of pain is important to detect negative impact on quality of life, such as discomfort and limitations on daily performances. Our study also indicates that pain is associated with missing teeth, suggesting that the treatment must be multidisciplinary, involving pharmacological, physical, psychological, and social care^20^.

Psychological limitations, such as sleeping and relaxing, reached 13 patients (5.62%). In approximately half (n = 6; 46.15%) of these patients, discomfort was expressed as the main related symptom. Conversely, Mendonça, et al.^11^ (2010) observed a higher prevalence rate (10.7%) considering the patient with missing teeth and hampered sleeping performance.

Oral health surveys must simultaneously consider the clinical, social, and psychological aspects that surround the patient because oral health conditions directly affect quality of life^21^, human behavior, and self-esteem^15^. Our study corroborates this information, showing that 40 patients (17.31%) expressed hampered maintenance of stable emotional status (OIDP6).

Even in the psychological performance, it is noteworthy the importance of using prosthesis/implant to reduce the impact of NSCL±P on activities related to smiling, laughing, and showing the teeth without getting embarrassed (OIDP5), and to maintaining the emotional state balanced without getting angry (OIDP6). Although the number of patients using prosthesis/implant was small (n = 37) in this study, no use had a significantly greater negative impact on OIDP5 and OIDP6 when compared to patients using prosthesis/implant.

Quality of life may be described as the progressive pathway in which a person enjoys daily activities, performances, and opportunities^15^. Consequently, the quality of life depends on external and internal factors, which may not be in harmony in NSCL±P patients, since these patients are often affected by the lack of social connection^22^. In our study, 13 (5.62%) patients reported hampered working performance (OIDP7), while 14 (6.06%) patients expressed difficulties for contacting other people (OIDP8). Mostly, aesthetic dissatisfaction, such as orofacial malformation, was the main associated complication.

OIDP is a social/dental tool used to quantify the oral health condition in relation to the associated impact on daily performances^2^. The quantified outcomes enable the identification of the main symptoms and complications related to the daily performances, reflecting physical, psychological, and social conditions^2^ ^,^ ^5^ ^,^ ^15^ at wide age ranges, making the screening of proper treatment planning also solid. Based on that, the OIDP was applied in this study.

The delay in the questionnaire application due to the length of questions and number of evaluated performances, as well as some difficulties of children and adolescents in understanding questions, are some limitations of this study.

Rehabilitation of NSCL±P patients is beyond the physical intervention, extending to the psychological and social environments from birth to adult ages.

## Conclusion

Almost half of the patients evaluated in this study showed negative impact of NSCL±P on the performance of daily activities. The high negative impact of NSCL±P on daily activities was associated with women. Physical and psychological damages figured as the most prevalent affected performances.
